# Popularising the Annual Report

**Published:** 1910-04-09

**Authors:** 


### POPULARISING THE ANNUAL REPORT.

### SOME SUGGESTIONS AND CRITICISM.

Year after year hospitals collect statistics with enormous labour, issue them in the clearest form, circulate them at considerable expense among their subscribers, and for their pains get the comforting assurance that the recipients, after a hasty glance to see whether their own names are correctly entered and how much their neighbours have contributed, consign the precious little pamphlet to the waste-paper basket. It is useless to blame the subscriber. Obviously the report, however admirable in substance, is not presented to him in a form which interests him. The figures, however lucid to an understanding mind, convey nothing to one unlearned in the intricacies of institution life. The evidences of rigid economy which the committee regard with pride are hidden from the eyes of the man in the street. And it must be admitted that, invaluable though these accurate compilations may prove to the student of hospital finance, and to the authorities of other hospitals, they fail altogether in the purpose for which they are intended—namely, the enlightenment of the citizen on whom the maintenance of the hospital must depend. Glancing hastily at the columns which sum up the expenditure, the verdict probably is " Astonishing what a lot of money is wasted in these big places. Here is £2,000 spent on butcher's meat, and yet everyone knows that the patients get scarcely anything but bread and milk."

#### Making the Figures Tell.

It is worth considering whether it might not be possible to bring the figures home to the minds of subscribers by affording them information which would help them to realise the institution as a series of households, bigger certainly than those of the ordinary subscribers, but not differing materially from these in their needs or relative expenditure. To descend into detail respecting every department of the institution every year would be not only far too costly, but also a trifle monotonous. It would, however, be quite within the compass of a brief report to single out each year some fresh aspect of the work and the cost it entails, bring it home to the minds of the ordinary reader by rendering comparison with his own household expenses possible, and thus lay the basis of a sound knowledge of institution management among the people who are to be relied on for furnishing the sinews of war. It goes without saying that the secretary who is prepared to write a report on these lines must so keep his accounts that he has all the necessary points at his finger-ends, or can procure them by the expenditure of a few hours' work on the part of his clerk. But the financial effect of inviting the public to take an intelligent interest in the working of their own concern is so striking that this small labour is likely to be repaid a thousand fold.

#### Food and Supplies.

The general condition of ignorance on the part of the public with regard to the patients' food, of which the remark quoted above is a specimen, must frequently have been brought to the notice of hospital workers. Let it be the first task of the annual report to dissipate this. As ordinarily presented, the report states the amount of the butcher's, the baker's, the milkman's, the grocer's bill for the whole establishment. These figures throw no light on the cost of the patient. The diet tables which are sometimes appended give more information, but they are not accompanied by any figures, so that the inquirer does not know how many patients fall into the different categories, nor how much per head these diets cost in practice. If the report is to aim at making the daily feeding of these sick people a reality, exciting sympathetic interest in their necessities and the manner in which they are provided for, it must convey the idea of a succession of groups, differing in their requirements, but all catered for on exact and frugal lines. The temptation to deal in averages must be avoided. The average cost of one person's food in an institution is not the actual cost of that of any one individual, and hence is devoid of interest to ordinary people who like concrete illustrations and what they call " facts."

#### Diet Facts!

To get at the facts, it is a good plan to take a specimen period and go through the accounts, separating the various classes of inmates whose board is on an identical basis, reckon up heads, estimate the expenditure incurred by each, and gather the whole into a connected statement. Taking a specimen day in the year, and mentioning the date, the report would go through the inmates of the institution class by class. First in order will come the patients, and these may be roughly divided into (*a*) those on milk diet, (*b*) those on simple diet, admitting broth or beef tea, but excluding meat, fish, or poultry, (*c*) those on ordinary diet, (*d*) those on special diet, including extras. The number of those on milk diet varies from about 3 to 12 per cent., and the cost is about 4d. a day, three pints a day being the usual allowance. The second class cost from 4d. to 8d. a day, according to the amount of beef tea ordered. Ordinary diet, embracing from 60 to 70 per cent. of the patients, costs about 10d. a day, or, if tea, sugar, and butter are not provided, 1d. or 1½d. less. Special diets need computing carefully, and may include fowl, rabbit, jelly, eggs, etc. The report would tabulate these facts somewhat as follows: On April 2 the ——— hospital contained 189 patients. Of these twelve were on milk diet, receiving three pints a day at a cost of 3¾d. Total, 3s. 9d. Forty-eight patients were on low diet, consisting of bread 10 oz., ¾d.; milk 1 pint, 1¼d.; beef tea 1 pint, 2d.; broth 1 pint, 2d.; cost, 6d. per day. Total, £1 4s. One hundred and twenty-two patients were on ordinary diet, consisting of bread, 10 oz., ¾d.; 6 oz. of roast mutton, 3d.; potatoes ½ lb., ¼d.; rice pudding, ¾d.; milk 1 pint, 1¼d.; soup or broth 1 pint, 2d.; tea, sugar and butter, 1½d.; sundries, ½d. Cost, 10½d. Total,

£5 6s. 9d. In addition there were seven patients on special diets, and the cost of diabetic foods for three of this number and of the fowl and eggs provided for the others brought the cost of their diets to 1s. 3d. each, or a total of 8s. 9d. Thus the patients' food on this specimen day cost £7 3s. 9d. The figures are adapted from the estimates in "Hospital Expenditure, The Commissariat" (Scientific Press), but it is understood that the whole value of such estimates would be their strict accuracy as compiled from the hospital accounts.

### The Nursing Department.

Proceeding to the other inmates of the institution, the nurses' home stands first in importance. The numbers of members of the staff in residence on this day should be given, and the meals provided enumerated, not omitting the arrangements for the night staff. The menu of the dinner and supper might be added, and the cost of the day's rations per head should be carefully computed, with its total cost appended. Next would follow the cost of rations for the medical and other officers if their numbers are sufficient to furnish a separate table, together with allowances for lunch. The number of male and female servants respectively would follow, with an estimate per head of cost of their board worked out from the actual figures. There certainly need be no mystery about the cost of food in the establishment, and the method we have suggested would help subscribers to understand exactly how the large sums are spent which they are called on to provide, and would remove many foolish misapprehensions as to the scale on which the household is managed. The total cost of food in the institution for one day thus analysed presents the most convincing answer to rumours of extravagance that could possibly be devised.

### The Consumption of Alcohol.

There are many other directions in which institutions find themselves exposed to criticism, and in almost all of these the annual report, properly edited, might convey the facts which are the only convincing answer to criticism. The use of alcohol is one of the most controversial points in hospital administration. It is usual to work out the amount used in the institution and present it in the form of an average on the number of beds. This way may be useful for purposes of comparison with other institutions, but it conveys nothing to the ardent temperance supporter of the local hospital. He wants to know how much is allowed to individuals. But to take one specimen day and note what amount of alcohol was used, and in what form, so many half-pints of beer among the porters, so many ounces of alcohol in the wards, would demonstrate to the critic the large extent to which the household were total abstainers, and the slight extent to which alcohol figures in medical treatment, for those who take the trouble to reckon out heads in conjunction with the amount of alcohol of every description used in institutions have long ago reached the conclusion that its use as a beverage for the staff of these establishments is actually negligible, and that its use in the wards is steadily decreasing.

### Coal.

Coal is an item which figures largely in the accounts, and its use is difficult to check. The annual report might do good service in computing the quantity used on an ordinary winter day, when all the fires are going at full strength; the daily amount of hard coal required for the engine-room, for cooking, laundry, and steam heating; the number of open fires kept going all day, or night and day; the weight of coal needed to replenish all the coal bunks, and the daily cost at the rate for which the coals were bought. This would set a chain of thought at work which would haunt the business man when he shovelled more coals on to his fire at home, and would keep his tongue from easy talk about hospital extravagance. The cost of coal at the London Hospital in 1908 was £5,308. Would it not stimulate the imagination and loosen the purse-strings of the hospital supporters to know how many tons of coal were used in one day, and in how many stoves and grates? The same holds good with regard to lighting. Could not the annual report of an institution vouchsafe some information about the number of lights required in the establishment, the rooms and wards to be lighted, and the measure of gas or electricity consumed during a specimen twenty-four hours in winter and summer?

### The Value of a Good Report.

There can be little doubt that reports worked out on the system of giving concrete information every year about some department of hospital activities would benefit not merely the subscriber but the actual workers themselves. There is an immense pleasure lying in the sphere of domestic economy if it can make itself a voice and testify to its own value. Want of method, want of interest follow inevitably on neglect, and the zeal stifled every year by official reticence would suffice to wake up a generous public to full appreciation of the triumphs of hospital finance in our voluntary institutions.

